# RNA-Binding Protein OsGRP3 Positively Regulates Rice Storability

**DOI:** 10.3390/plants15030464

**Published:** 2026-02-02

**Authors:** Dongxu Wen, Naibin Zhang, Jiahui Shi, Yuqin Tang, Chiyu Li, Long Wang

**Affiliations:** 1 College of Biology, Chongqing Research Institute, Hunan University, Changsha 410082, China; dongxu0906@hnu.edu.cn (D.W.); chunbinbin@hnu.edu.cn (N.Z.); sjh0914@hnu.edu.cn (J.S.); yuqintang@139.com (Y.T.); 2 College of Longping Agricultural, Hunan University, Changsha 410082, China; 3 Hunan Provincial University Key Laboratory of the Fundamental and Clinical Research on Neurodegenerative Diseases, Changsha Medical University, Changsha 410219, China; 4 School of Life Sciences, Central South University, Changsha 410078, China; 5 State Key Laboratory of Hybrid Rice, Hunan Academy of Agricultural Sciences, Changsha 410125, China

**Keywords:** OsGRP3, long lived mRNA, seed vigor, rice storability

## Abstract

Seed aging during storage represents a major challenge to global food security and germplasm resource conservation. Long-lived mRNAs, which are crucial for initiating germination after storage, have poorly understood regulatory mechanisms governing their stability. In this study, we identify the RNA-binding protein OsGRP3 as a key positive regulator of rice storability. Initially, we demonstrated that *Arabidopsis* AtGRP7 enhances seed vigor following aging. Phylogenetic analysis identified OsGRP3 as its closest rice homolog. Two independent *OsGRP3*-overexpression lines showed markedly improved germination rates and seed viability after extended artificial aging. Physiological assessments indicated that OsGRP3 mitigates aging-related damage, as evidenced by reduced malondialdehyde (MDA) levels and electrolyte leakage, consistent with better membrane integrity. RNA-seq analysis revealed that *OsGRP3* overexpression attenuated the transcriptional disruption induced by aging. Moreover, under non-stress conditions, OsGRP3 directs a transcriptional program involving 404 genes implicated in DNA replication, gluconeogenesis, and essential amino acid metabolism. This reprogramming correlates with a state of heightened stress preparedness, exhibiting a pattern of correlated transcriptional regulation. Our findings establish OsGRP3 as a conserved RNA-binding protein that enhances seed storability, and offer a promising genetic target for improving storage tolerance in rice.

## 1. Introduction

Rice, a staple crop supporting more than half of the world’s population, is fundamental to global food security [1]. However, prolonged storage leads to a marked decline in seed vigor and a deterioration in grain quality, undercutting both nutritional value and agricultural productivity, a challenge particularly acute in tropical and subtropical regions [2]. Improving storage tolerance is therefore essential to securing grain preservation and sustainable seed use, establishing it as a priority trait in modern seed breeding. Uncovering genetic resources related to storage tolerance and deciphering the molecular basis of seed aging are thus critical for minimizing postharvest losses and safeguarding long-term food security [3,4].

During seed aging, a series of physiological and biochemical alterations take place, such as the inhibition of enzyme activity, accumulation of harmful metabolites, decline in antioxidant capacity, lipid peroxidation, biomembrane disruption, and damage to biological macromolecules including nucleic acids and proteins [2,5,6]. It is widely established that the production and dysregulation of reactive oxygen species (ROS) serve as the principal drivers of seed deterioration [7]. During the later stages of storage, for instance, excessive ROS accumulation triggers lipid peroxidation, inflicts macromolecular damage, and results in DNA strand breaks [2,8,9,10]. Beyond oxidative damage, the stability of long-lived RNAs, a class of RNA molecules preserved in seeds that are crucial for early germination, also considerably affects seed storability [11,12,13,14]. Nevertheless, the protective mechanisms that maintain the integrity of these long-lived RNAs throughout the aging process remain largely unknown.

Glycine-Rich RNA-Binding Protein 7 (GRP7), a member of the glycine-rich RNA-binding protein (GR-RBP) family, specifically binds RNA and participates in post-transcriptional regulatory processes, including alternative splicing and translation, thereby modulating plant adaptation to environmental stresses [15,16,17]. Recent studies have revealed that GRP7 can also bind RNA extracellularly and contribute to the stability of extracellular RNAs (exRNAs) in the apoplastic fluid [18]. In rice, expression of its homolog OsGRP1A increases steadily during seed storage [19], raising the question of whether OsGRP enhances seed storage tolerance via RNA regulation.

Here, building on the preliminary finding that *Arabidopsis* AtGRP7 positively regulates seed vigor, we hypothesized that its closest rice ortholog, OsGRP3, might play a conserved role in governing seed storability. To test this hypothesis, we aimed to functionally characterize OsGRP3 in rice. Our objectives were to determine whether OsGRP3 overexpression enhances tolerance to artificial aging, assess its impact on cellular damage, and elucidate the global transcriptional changes it orchestrates. Our results confirm that OsGRP3 is a positive regulator of seed storability. The transcriptomic and physiological data suggest that this function is mediated primarily through the regulation of DNA replication, stabilization of amino acid-related mRNAs, and starch content, which together enhance the resilience of seeds to aging stress.

## 2. Results

### 2.1. AtGRP7 Positively Regualtes Seed Vigor

**Figure 1:**
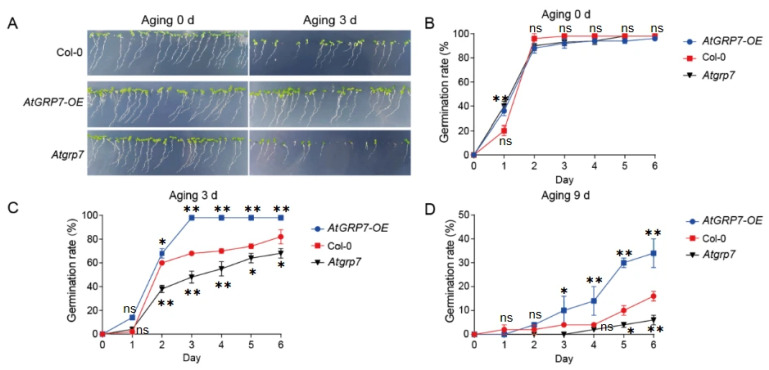
AtGRP7 modulates seed germination and aging tolerance in *Arabidopsis*. (**A**) Phenotypic observation of seed germination in Col-0, *AtGRP7-OE* (*AtGRP7*-overexpression), and *atgrp7* lines after 0 and 3 days of artificial aging. (**B**) Dynamic changes in germination rates of Col-0, *AtGRP7*-*OE*, and *atgrp7* seeds without prior aging (0-day aging). (**C**) Germination rate dynamics of Col-0, *AtGRP7*-*OE*, and *atgrp7* seeds following 3 days of aging. (**D**) Germination rate dynamics of Col-0, *AtGRP7*-*OE*, and *atgrp7* seeds after 9 days of severe aging. The data demonstrate the critical function of AtGRP7 in enhancing seed storability and maintaining germination capacity under prolonged aging treatment. All assessments were independently repeated three times with similar results. One-way ANOVA with LSD test, * *p* < 0.05; ** *p* < 0.01; ns means not significant.

To further investigate the role of the RNA-binding protein AtGRP7 in maintaining seed vigor, we conducted systematic artificial accelerated aging experiments using wild-type (Col-0), *AtGRP7*-overexpression (*AtGRP7-OE*), and *atgrp7* mutant seeds [15]. Germination rate analysis showed that all genotypes exhibited similarly high germination levels under untreated conditions (0 d; Figure 1A,B), indicating no significant differences in initial seed viability among the genotypes. However, after 3 days of artificial aging treatment, distinct phenotypic differences emerged: the *AtGRP7-OE* lines maintained strong germination capacity and normal seedling establishment, comparable to the non-aged control group, whereas the *atgrp7* mutant showed the most severe decline in viability, with significantly reduced germination rates (Figure 1A,C). When the aging period was extended to 9 days, the genotypic differences became more pronounced: *AtGRP7-OE* seeds consistently exhibited significantly higher germination rates than the wild-type control, while the *atgrp7* mutant displayed the lowest germination level among all lines (Figure 1D). These time-course experimental results demonstrate that AtGRP7 actively participates in maintaining seed vigor under artificial aging stress and functions as a positive regulator in this process.

### 2.2. The Homologous Gene of AtGRP7, OsGRP3, Is Expressed in Rice

**Figure 2:**
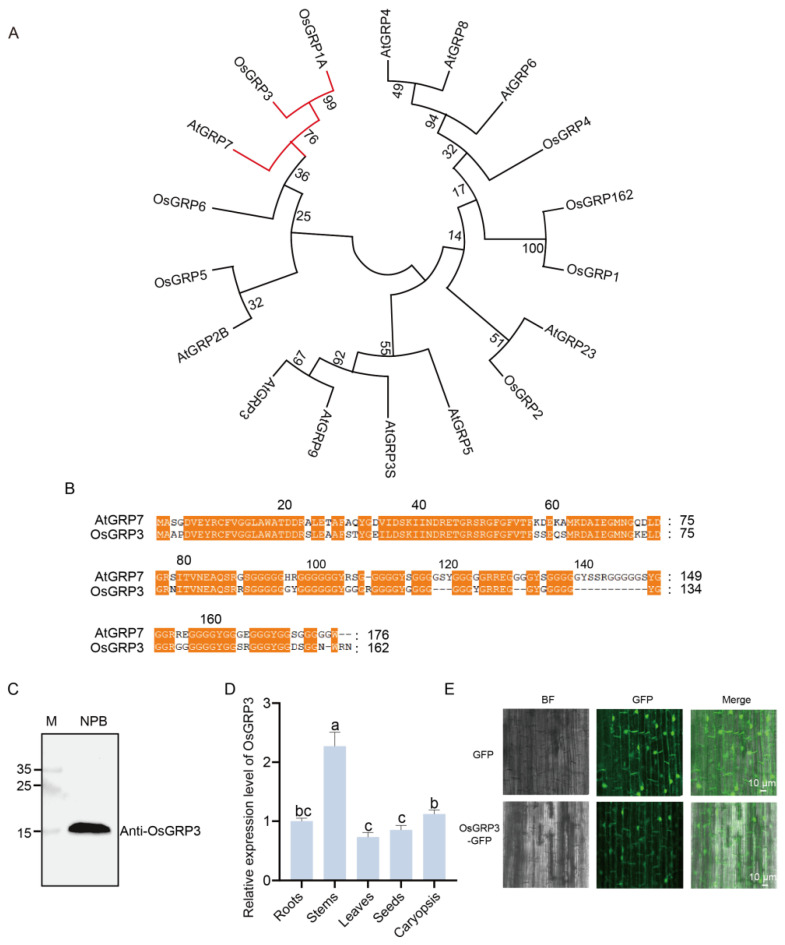
Phylogenetic relationship, sequence alignment, expression pattern, and subcellular localization of rice OsGRP3. (**A**) Phylogenetic tree of GRP family members from rice and *Arabidopsis*. Red branch indicates close evolutionary relationship between AtGRP7 and OsGRP3. Branch support values from 1000 bootstrap replicates are shown at nodes. (**B**) Amino acid sequence alignment of AtGRP7 and OsGRP3. Conserved domains, indicative of potential functional similarity, are marked by orange boxes. (**C**) Immunoblot validation of the OsGRP3 protein. Lane M represents the protein molecular weight marker, and Nip serves as the negative control. A specific band for OsGRP3 confirms its detection. (**D**) Relative OsGRP3 protein expression levels in different rice organs. The ‘Root’ sample was designated as the calibrator (set to a value of 1), and all other expression levels are presented relative to this baseline. *OsActin2* and *PP2A* were selected as reference genes, and similar results were obtained with both. Different letters indicate significant differences (*p* < 0.05, ANOVA with Tukey’s test, *n* = 3). (**E**) Subcellular localization of the OsGRP3-GFP fusion protein (lower panel) and the GFP-only control (upper panel) in rice root cells. Images from left to right represent the bright field (BF), GFP green fluorescence channel, and the merged image.

Phylogenetic analysis revealed that OsGRP3 shares the closest evolutionary relationship with AtGRP7 in rice (Figure 2A), suggesting potential functional conservation between these two proteins. Further protein sequence alignment demonstrated multiple highly conserved regions between OsGRP3 and AtGRP7 (Figure 2B, orange highlights), particularly within the N-terminal domain, indicating that OsGRP3 likely maintains structural and functional characteristics similar to AtGRP7.

To determine whether OsGRP3 is expressed in seeds, we employed an endogenous antibody for detection. A single clear band of the expected molecular weight was detected in the Nip cultivar (Figure 2C), confirming that OsGRP3 is normally expressed in rice seeds. Tissue-specific expression analysis revealed distinct expression patterns, with the highest abundance in stems, followed by caryopses and roots, while relatively lower levels were observed in leaves and seeds (Figure 2D). Subcellular localization experiments revealed that the GFP signal from the OsGRP3-GFP fusion protein was observed in the nucleus and at the cell periphery, a pattern consistent with a nucleo-cytoplasmic localization, that would be the predicted location for an AtGRP7 homologue (Figure 2E). Collectively, OsGRP3 is a structural homolog of AtGRP7 in rice, and its expression in seeds suggests a potential role in seed storability regulation.

### 2.3. OsGRP3 Positively Regulates Seed Vigor

**Figure 3:**
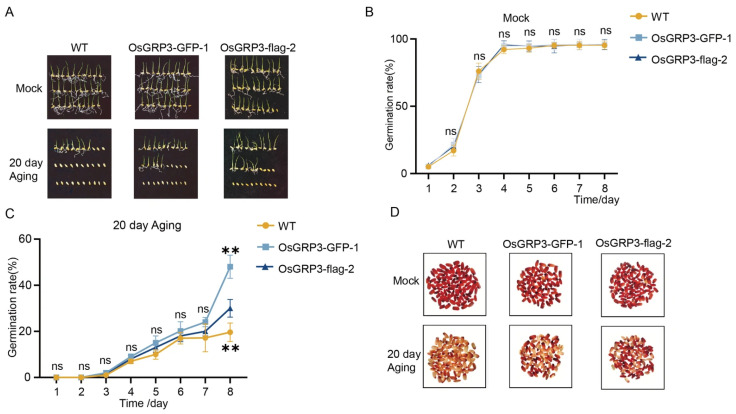
Overexpression of *OsGRP3* enhances seed aging tolerance in rice. (**A**) Germination phenotypes of wild-type (WT), *OsGRP3-GFP-1*, and *OsGRP3-flag-2* seeds under non-aged (Mock) and 20-day artificial aging conditions. (**B**) Daily germination rates without aging treatment. All genotypes maintain high germination capacity under normal storage. (**C**) Daily germination rates after 20-day aging. WT seeds show significantly reduced germination compared to overexpression lines. Data are mean ± SD (*n* = 3, ** *p* < 0.01; ns means not significant ). (**D**) TTC staining phenotypes of WT, *OsGRP3-GFP-1*, and *OsGRP3-flag-2* seeds under non-aged (Mock) and 20-day artificial aging conditions.

To elucidate the biological function of OsGRP3 in maintaining rice seed vigor, we successfully generated two independent overexpression lines, *OsGRP3-GFP-1* and *OsGRP3-flag-2* (Figure S1B). Under normal growth conditions, the germination rates of transgenic lines and wild-type seeds were comparable (Figure 3A,B), indicating that OsGRP3 overexpression does not affect the normal seed germination process. Following 20 days of artificial accelerated aging treatment, significant differences emerged among genotypes. Wild-type seeds showed a marked decline in germination rate, while both overexpression lines maintained higher germination capacity throughout the observation period (Figure 3A,C). This difference became particularly pronounced during the later stages of treatment, suggesting that OsGRP3 overexpression effectively alleviates the damage caused by aging stress on seed germination capability. In addition, we assessed metabolic activity in seed embryos using TTC staining. The results revealed that after aging treatment, the embryos of overexpression lines exhibited more intense red staining (Figure 3D), indicating preserved dehydrogenase activity and cellular metabolic function. This indicates that OsGRP3, similar to AtGRP7, positively regulates seed vigor in rice.

We have duly noted the difference in storage tolerance between *japonica* and *indica* rice, and conducted an analysis focusing on the genetic variation of the OsGRP3 between the two subspecies. Analysis of the OsGRP3 sequence revealed no significant variation in its promoter region. In the coding region, however, we identified two site-specific mutations that result in amino acid alterations. Notably, these mutations are not differentiated between the *indica* and *japonica* subspecies, as both variant types are present in each group (Figure S2). This further suggests that OsGRP3 may not contribute to the difference in storage tolerance between *indica* and *japonica* rice.

### 2.4. OsGRP3 Overexpression Sustains Seed Storability by Alleviating Membrane Damage During Prolonged Aging

**Figure 4:**
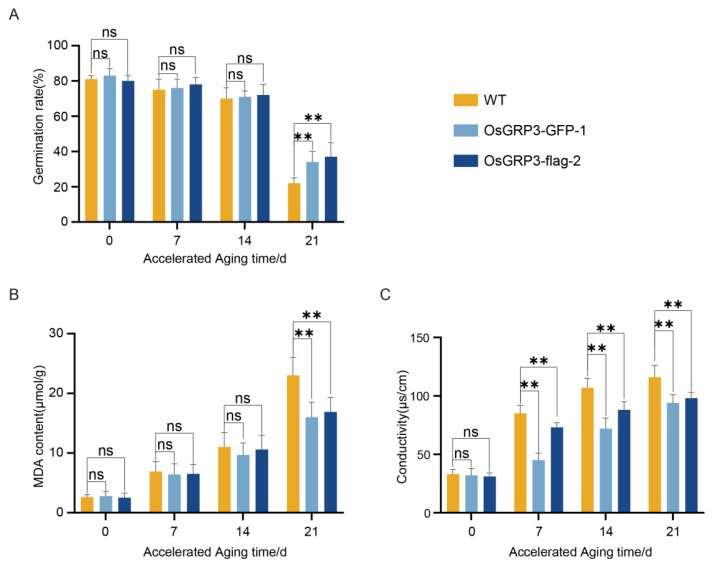
Effects of *OsGRP3* overexpression on germination rate, malondialdehyde (MDA) content, and electrolyte leakage in rice seeds during accelerated aging. (**A**) Germination rates of wild-type (WT), *OsGRP3-GFP-1*, and *OsGRP3-flag-2* seeds after 0, 7, 14, and 21 days of accelerated aging (** *p* < 0.01; mean ± SD; *n* = 3, One-way ANOVA with Tukey’s test). (**B**) MDA content in WT, *OsGRP3-GFP-1*, and *OsGRP3-flag-2* seeds during accelerated aging. MDA levels showed no significant differences at 0, 7, and 14 days (mean ± SD; *n* = 3, One-way ANOVA with Tukey’s test). (**C**) Electrolyte leakage measurements in WT, *OsGRP3-GFP-1*, and *OsGRP3-flag-2* seeds during accelerated aging. No significant difference was detected at day 0 (mean ± SD; *n* = 3, One-way ANOVA with Tukey’s test); ** *p *< 0.01; ns means not significant.

To evaluate the effect of OsGRP3 on seed storability, we monitored the germination performance and membrane integrity of wild-type (Nip) and two independent OsGRP3-overexpression lines (*OsGRP3-GFP-1* and *OsGRP3-flag-2*) during a 21-day accelerated aging process. Initially (0 d), all genotypes exhibited similarly high germination rates (>95%), indicating comparable seed viability (Figure 4A). As aging progressed, wild-type seeds showed a continuous and significant decline in germination capacity, decreasing to approximately 40% by day 21. In contrast, both OsGRP3-overexpression lines maintained significantly higher germination rates (approximately 75–80%) at the later stages (Figure 4A). These results demonstrate that OsGRP3 overexpression confers sustained seed storability under extended aging stress.

To determine whether this maintained vigor was associated with reduced oxidative damage to cellular membranes, we quantified malondialdehyde (MDA) levels, a biomarker of lipid peroxidation [20]. Throughout the aging period, wild-type seeds exhibited a time-dependent increase in MDA accumulation. In contrast, both overexpression lines showed significantly lower MDA levels at all time points, particularly at day 21 (Figure 4B). This indicates that OsGRP3 overexpression attenuates membrane lipid peroxidation during seed aging.

We further assessed membrane integrity by measuring electrolyte leakage. Wild-type seeds displayed a progressive increase in ion leakage, reflecting cumulative membrane damage. Meanwhile, electrolyte leakage measurements, which reflect membrane integrity, demonstrated significantly lower rates in overexpression lines compared to wild-type during the entire aging process (Figure 4C). These results are consistent with the observed reduction in MDA levels and together indicate that OsGRP3 overexpression is associated with better maintenance of membrane integrity under aging stress [21].

### 2.5. OsGRP3 Overexpression Preserves Starch Content and Cooking Quality in Aged Seeds

**Figure 5:**
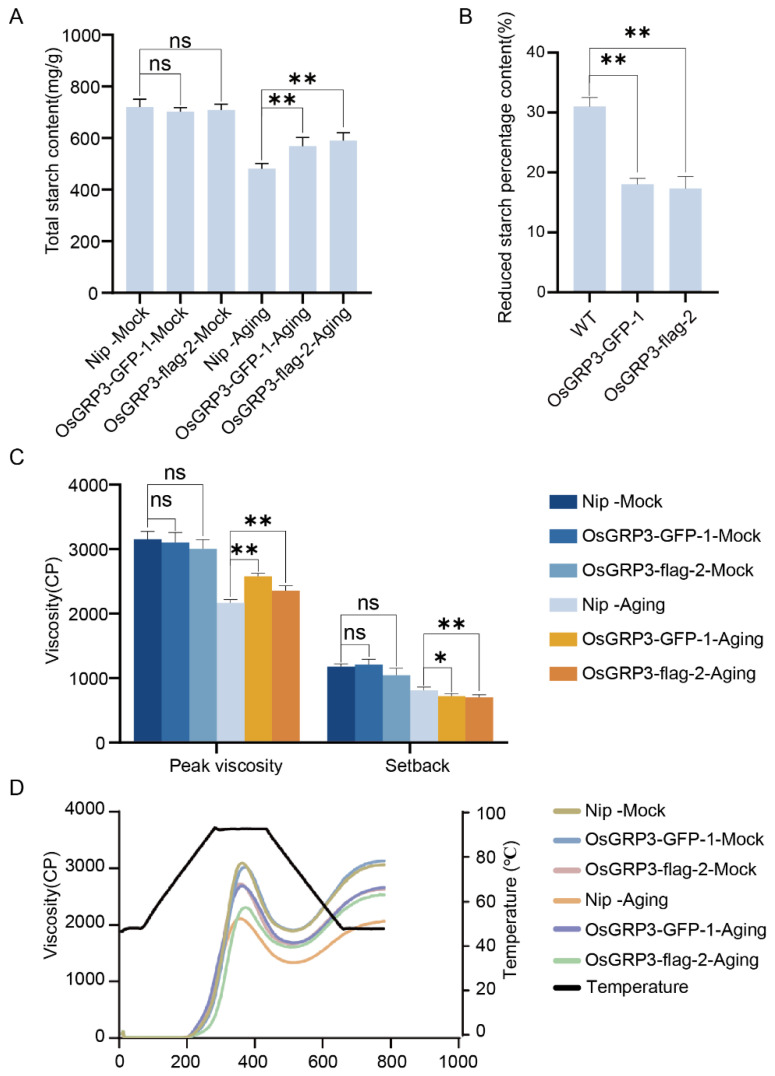
Effects of *OsGRP3* overexpression on starch content, starch reduction percentage, and pasting properties in rice seeds. (**A**) Total starch content under non-aged (Mock) and aged conditions. ns: not significant; ** *p* < 0.01; ns means not significant. Data are mean ± SD (*n* = 3, one-way ANOVA with Tukey’s test). (**B**) Statistical analysis of the reduced starch percentage in Nip, *OsGRP3-GFP-1*, and *OsGRP3-flag-2* seeds (** *p* < 0.01; mean ± SD; *n* = 3, one-way ANOVA with Tukey’s test). (**C**) Pasting properties (peak viscosity and setback value) under Mock and aged conditions. After aging, Nip exhibits significantly reduced peak viscosity compared to overexpression lines (** *p* < 0.01, * *p* < 0.05; mean ± SD; *n* = 3, one-way ANOVA with Tukey’s test). (**D**) Pasting viscosity curves of starch from Nip, *OsGRP3-GFP-1*, and *OsGRP3-flag-2* seeds under non-aged (Mock) and aged conditions.

We next examined whether the improved storability extended to seed composition and culinary traits. Under non-aged (Mock) conditions, no significant differences in total starch content were observed between wild-type and transgenic lines (Figure 5A). However, after accelerated aging treatment, wild-type seeds exhibited a marked reduction in starch content, whereas both overexpression lines retained significantly higher levels (Figure 5A). Quantitative analysis confirmed that the percentage reduction in starch content after aging was significantly lower in the overexpression lines compared to wild-type (Figure 5B). These findings suggest that OsGRP3 overexpression mitigates starch degradation during the aging process.

Functional analysis of starch pasting properties revealed that prior to aging, all genotypes displayed comparable peak viscosity and setback values (Figure 5C). Following aging, however, wild-type seeds showed a substantial decline in peak viscosity and an increase in setback—indicators of reduced cooking quality and increased retrogradation. The overexpression lines, in contrast, maintained significantly higher peak viscosity and lower setback values after aging (Figure 5C). The complete pasting curves further demonstrated that the transgenic lines better preserved their viscosity profiles under aging stress (Figure 5D).

These results demonstrate that the OsGRP3-overexpression phenotype encompasses not only prolonged seed viability but also the preservation of starch content and key functional properties determining cooking quality, highlighting its potential for improving post-harvest traits in rice.

### 2.6. OsGRP3 Overexpression Alters the Transcriptional Program of Aging-Associated Genes

To elucidate the function of OsGRP3 in rice seed development and aging response, we performed RNA-seq analysis on mature seeds of wild-type (Nip) and OsGRP3 overexpression lines. The experimental design included untreated control groups and 15-day artificial accelerated aging treatment groups, with three biological replicates per condition.

Principal component analysis (PCA) revealed tight clustering of biological replicates across all materials, both before and after aging, confirming high reproducibility of the transcriptome data (Figure S3). In wild-type seeds, aging stress induced extensive transcriptional reprogramming, with 5117 genes up-regulated and 4433 down-regulated (Figure S4A, Table S3). Notably, even under non-stress conditions, OsGRP3 overexpression alone modulated the expression of 916 genes (332 up, 584 down; Figure S4B, Table S4). Compared to wild-type, OsGRP3-overexpressing lines exhibited a more attenuated transcriptomic shift upon aging, with 4218 genes up-regulated and 3817 down-regulated (Figure S4C, Table S5), suggesting that OsGRP3 mitigates mRNA instability during seed aging.

**Figure 6:**
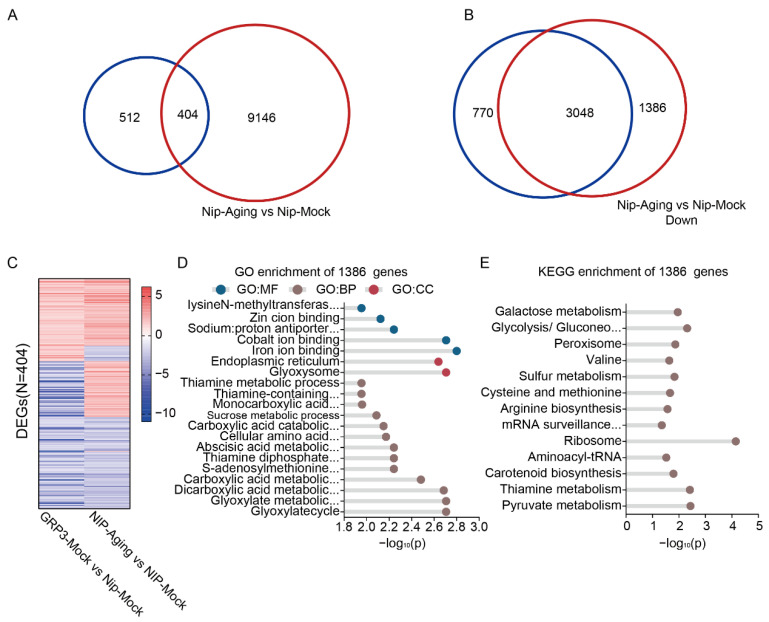
Transcriptomic analysis of seed aging and OsGRP3 overexpression in rices. (**A**) Venn diagram showing the overlap of DEGs between the OsGRP3-Mock vs. Nip-Mock comparison (blue circle) and the Nip-Aging vs. Nip-Mock comparison (red circle). (**B**) Venn diagram showing the overlap of down-regulated DEGs between the OsGRP3-Aging vs. OsGRP3-Mock comparison (blue circle) and the Nip-Aging vs. Nip-Mock comparison (red circle). (**C**) Heatmap displaying the expression patterns of alternative splicing events for the 404 common DEGs identified in both the OsGRP3-Mock vs. Nip-Mock and Nip-Aging vs. Nip-Mock comparisons. The color gradient represents expression level differences, visually presenting the consistent expression patterns of these common DEGs under both conditions. (**D**) GO enrichment analysis of the 1386 unique DEGs. Significantly enriched terms are presented from three categories: Molecular Function (GO:MF), Cellular Component (GO:CC), and Biological Process (GO:BP). The x-axis shows the −log_10_(P). (**E**) KEGG pathway enrichment analysis of the 1386 unique DEGs. The x-axis shows the −log_10_(P).

We further identified a significant overlap between the 916 genes differentially expressed in OsGRP3-overexpressing lines under non-stress conditions and those altered in aged Nip seeds, with 404 genes common to both sets (Figure 6A). These overlapping genes were significantly enriched in key biological pathways such as DNA replication, gluconeogenesis, and essential amino acid metabolism (Figure S4D,E). The consistent expression patterns of these genes under both conditions (Figure 6C) indicate that OsGRP3 may associated with a potential pre-adaptation transcriptional signature, which may enhance the seeds’ ability to cope with subsequent aging stress.

Our transcriptomic analysis revealed that OsGRP3 overexpression profoundly alters the seed’s transcriptional landscape during aging. Comparison of downregulated genes identified 1386 genes that were significantly suppressed in aged wild-type seeds but maintained in aged OsGRP3-overexpressing seeds (Figure 6B, Tables S6 and S7). Comprehensive functional enrichment analysis of these preserved genes demonstrated that they are strongly associated with multiple critical processes including glyoxylate metabolic, carboxylic acid metabolic, sucrose metabolic, glycolysis/gluconeogenesis, peroxisome function, arginine biosynthesis, mRNA surveillance pathway, pyruvate metabolism, ribosome, and endoplasmic reticulum function (Figure 6D,E, Tables S6 and S7). Notably, genes encoding iron-binding proteins, which are critical for regulating redox homeostasis and preventing Fenton reactions, were enriched among the preserved transcripts. Similarly, endoplasmic reticulum-associated genes involved in protein folding and quality control were also maintained. This suggests that OsGRP3 overexpression preserves cellular detoxification capacity and protein homeostasis under aging stress. The preserved transcripts associated with glycolysis/gluconeogenesis and pyruvate metabolism are essential for providing energy during early germination, while those involved in mRNA surveillance contribute to maintaining mRNA stability. Additionally, the maintenance of peroxisome function, carotenoid biosynthesis, and sulfur metabolism pathways likely helps mitigate oxidative damage. The coordinated preservation of these diverse functional categories indicates that OsGRP3 sustains seed storability not merely by attenuating general mRNA degradation, but by selectively preserving a network of mRNAs critical for energy metabolism, redox homeostasis, protein quality control, and stress defense, all of which are essential for successful germination initiation.

## 3. Discussion

Our study establishes that OsGRP3, the rice ortholog of *Arabidopsis* AtGRP7, enhances seed storability by the modulation of transcriptional profiles related to stress adaptation [22,23,24,25]. We demonstrated that OsGRP3 coordinates a precise transcriptional program, which pre-configures the seed to better withstand aging stress. This is evidenced by the significant overlap of 404 differentially expressed genes between OsGRP3-overexpressing seeds and aged wild-type seeds, suggesting that OsGRP3 activation mimics a state of stress preparedness. The enrichment of these genes in DNA replication, gluconeogenesis, and amino acid metabolism implies that OsGRP3 pre-activates essential metabolic and repair pathways, thereby preserving viability during storage. These findings position OsGRP3 as a key regulator that primes seeds against aging stress. Furthermore, the functional conservation between OsGRP3 and AtGRP7 highlights the potential for RNA-binding proteins to play an evolutionarily conserved role in regulating seed aging across plant species.

Notably, OsGRP3 maintains the expression of 1386 genes normally suppressed during aging in wild-type seeds. These genes, involved in ribosome biogenesis, arginine biosynthesis, and glycolysis/gluconeogenesis, are essential for seed resuscitation. Given the conserved RNA-binding domain between OsGRP3 and AtGRP7 [17], and OsGRP3’s ability to bind heat stress-responsive mRNAs [26], we speculate that OsGRP3 stabilizes key transcripts by recognizing similar cis-elements. Based on the conserved nature of these pathways as targets of long-lived RNAs [27,28], we propose a working model where OsGRP3 stabilizes critical mRNAs, forming a protected RNA reservoir that ensures rapid recovery upon imbibition.

Furthermore, as an RNA-binding protein, OsGRP3 likely exerts its role in enhancing seed storability through specific molecular interactions. While the precise partners of OsGRP3 in rice seeds remain to be fully elucidated, insights can be drawn from its *Arabidopsis* homolog, AtGRP7. AtGRP7 and OsGRP3 have been demonstrated to interact with numerous mRNA targets, influencing their stability and alternative splicing [15,26]. They also physically associates with core spliceosome components, such as U1-70K, to modulate pre-mRNA processing. Therefore, a plausible hypothesis is that OsGRP3 may similarly bind to and regulate the transcripts of key genes involved in oxidative stress response, seed storability, and starch metabolism. The broad transcriptional reprogramming observed in our RNA-seq data supports this notion.

Transcriptomic changes mediated by OsGRP3 explain the observed physiological phenotypes. Preserved expression of peroxisome, carotenoid, and redox homeostasis genes mitigates oxidative damage, aligning with reduced MDA levels and electrolyte leakage. Similarly, sustained glycolysis/gluconeogenesis gene expression supports starch stability, correlating with higher starch content and improved pasting properties after aging. These links confirm that OsGRP3 enhances storability by selectively maintaining genes critical for key physiological traits [29,30].

Reduced malondialdehyde accumulation and electrolyte leakage in OsGRP3 overexpression lines demonstrate enhanced membrane integrity maintenance [31,32], while preserved cooking quality and starch properties highlight the practical implications of our findings for grain storage. It should be acknowledged that the functional analysis in this study is based on constitutive overexpression lines, which may present certain limitations. In conclusion, our work establishes OsGRP3 as a regulator in the seed aging response, and we propose that it may operate through its role in a correlated transcriptional regulation and stabilization of long-lived mRNAs. The maintenance of a long-lived RNA reservoir, as proposed in our model, would represent a sophisticated strategy for preserving seed viability during storage, offering fundamental insights in agricultural contexts.

## 4. Materials and Methods

### 4.1. Plant Material Generation

The full-length coding sequence of OsGRP3 was cloned into the pCAMBIA1390 vector, which was controlled by the ubiquitin promoter and had a GFP or FLAG tag at the C-terminal. This construct was introduced into the Agrobacterium tumefaciens EHA105 strain and was introduced into Nipponbare (Nip) through Agrobacterium-mediated transformation [33]. T1 seeds were harvested from T0 plants and subjected to another selection for hygromycin resistance. We performed another round of hygromycin selection using T1 seeds and obtained homozygous T2 seeds. All subsequent experiments used these homozygous T2 seeds to ensure trait stability. We then validated the storage-tolerant phenotype using two independent transgenic lines expressing GRP3-GFP and GRP3-flag, which yielded consistent results (Figure S1A,B). Given that the two plasmid backbones are identical except for the tag, we analyzed one GRP3-GFP line and one GRP3-flag line for the subsequent study.

The AtGRP7 overexpression (*AtGRP7-OE*) line and the *atgrp7* T-DNA insertion knockout mutant (*atgrp7*) were obtained from previously established and characterized materials [15]. All transgenic and mutant lines are in the Col-0 background. The transgenic lines used in our experiments are homozygous, as confirmed by genotyping and consistent phenotypic screening.

### 4.2. Phylogenetic Analysis

To precisely identify the rice ortholog of Arabidopsis AtGRP7, a comprehensive phylogenetic analysis was conducted within the GRP families of both species. All annotated GRP protein sequences from rice and Arabidopsis were retrieved from the MSU (https://rice.uga.edu/ (accessed on 16 January 2023)) and TAIR (https://www.arabidopsis.org/ (accessed on 20 April 2024)) databases, respectively. Multiple sequence alignment was performed using the MUSCLE algorithm in MEGA11, followed by manual optimization. The optimal amino acid substitution model (Jones–Taylor–Thornton model) was selected based on the Bayesian Information Criterion (BIC). A maximum likelihood phylogenetic tree was constructed, and branch robustness was assessed using 1000 bootstrap replicates.

### 4.3. Haplotype Identification Across the OsGRP3 Coding Region

The coding sequence (CDS) of OsGRP3 was selected for SNP identification using the RFGB v2.0 database [34]. The Nip genome was used as the reference genome. First, the gene ID of OsGRP3 was input, and then the gene region was selected, with loci having an allele frequency greater than 0.05 being filtered. The screening range covered the entire 3000 Rice Genomes (3K-RG) dataset.

### 4.4. Seed Germination Assay

For each seed sample, three biological replicates of 30 seeds each were set up. Seeds were evenly placed on 10 × 10 cm square grid dishes lined with two layers of filter paper, and moistened with an adequate volume of ddH2O. The dishes were then incubated in a growth chamber set at 30 °C under low-light conditions. The day following the start of incubation was designated as day 1 of germination. Germination rates were recorded daily at a fixed time. The assay was terminated on day 8, by which point the final germination rate was recorded, as no further significant change in germination percentage was typically observed beyond this time point [35,36].

### 4.5. Subcellular Localization Analysis

Transgenic rice plants stably expressing pUbi: OsGRP3-GFP and pUbi: GFP were utilized in this study. The harvested seeds were subjected to germination culture at a constant temperature of 26 °C for 4 days. The GFP fluorescence signals in the root organs were then visualized and analyzed using a confocal laser scanning microscope [37].

### 4.6. TTC Staining Assay

Rice seeds were first immersed in distilled water at 30 °C for 4 h to allow full imbibition. Subsequently, 100 seeds were randomly selected and completely immersed in a 0.5% (*w*/*v*) 2, 3, 5-triphenyltetrazolium chloride (TTC) solution in a Petri dish. The dish was sealed and incubated in the dark at 30 °C for 30 min. After incubation, the TTC solution was discarded, and the seeds were gently rinsed twice with distilled water. Staining of the embryos was immediately observed under a stereomicroscope. Seed viability was assessed based on the extent of red coloration in the embryonic tissues [38].

### 4.7. Treatment for Accelerating After-Ripening of Arabidopsis Seeds

To simulate natural seed aging within a shortened experimental timeframe, an internationally established artificial accelerated aging protocol was employed. Arabidopsis seeds with uniform initial moisture content and viability were used. An appropriate amount of seeds was evenly distributed in small Petri dishes. The dishes were sealed with plastic film, which was perforated uniformly with a fine needle to allow gas exchange. All dishes were then placed in a growth chamber maintained at 40 °C and 100% relative humidity for aging treatments of 0, 1, 2, and 3 days [39]. Three biological replicates were included for each treatment group.

### 4.8. Treatment for Accelerated Aging of Rice Seeds

To rapidly evaluate differences in storage tolerance among rice accessions, an artificial accelerated aging assay was employed using high-temperature and -humidity conditions. Freshly harvested, physiologically mature rice seeds were subjected to aging in an environmental chamber maintained at 42 °C and 85% relative humidity for 0, 5, 10, 15, and 20 days [13,40]. Following treatment, representative seeds from each time point were selected based on plumpness and the absence of visible damage, mold, or germination. The seeds were surface-sterilized in 10% (*v*/*v*) sodium hypochlorite solution for 40 min and subsequently rinsed thoroughly with ddH2O 3–5 times before further use.

### 4.9. Total Starch Content Assay

The total starch content was determined using an enzymatic hydrolysis method. In brief, the dried and ground rice seed samples were oscillated and centrifuged with 80% ethanol to remove free sugars. The precipitate was gelatinized at high temperature and then hydrolyzed by thermostable α-amylase and amyloglucosidase at 50 °C to release glucose. The glucose content in the hydrolysate was measured spectrophotometrically at 510 nm using the glucose oxidase peroxidase reagent. The total starch content was calculated by multiplying the glucose content by a conversion factor of 0.9 [41]. This method ensures complete and specific conversion of starch to glucose, avoiding interference from other polysaccharides, and has been widely applied in cereal starch analysis.

### 4.10. Pasting Property Measurement

The pasting properties of rice starch were determined using a Rapid Visco Analyzer. A mixture of 3 g starch and 25 g distilled water was prepared in an RVA testing canister (Perten, Stockholm, Sweden). The testing profile was set as follows: held at 50 °C for 1 min, heated uniformly to 95 °C over 8 min, maintained at 95 °C for 2.5 min, cooled uniformly to 50 °C over 8 min, and finally held at 50 °C for 2 min. The mixture was stirred at 60 r/min for the first 10 s, followed by continuous stirring at 160 r/min for the remainder of the test. The following parameters were recorded: peak viscosity, trough viscosity, final viscosity, pasting temperature, breakdown, and setback. The complete set of pasting property data obtained from this analysis is available in Supplementary Table S2. All viscosity values are expressed in Rapid Visco Units.

### 4.11. Texture Profile Analysis

Rice grains were debusked and polished. The resulting white rice was rinsed three times with distilled water and subsequently soaked in distilled water for 30 min. After soaking, the rice was cooked with approximately 1.3-fold (*v*/*w*) distilled water for 40 min, followed by a 20-min equilibration period. The cooked rice was cooled to room temperature before analysis. Texture profile analysis was performed using a texture analyzer equipped with a 36R cylindrical probe. The instrument was calibrated for height and force prior to measurement. Three intact rice grains were placed on the platform for each test. The testing parameters were set as follows: pre-test speed, 10 mm/s; test speed, 0.5 mm/s; post-test speed, 5 mm/s; compression ratio, 70%; and trigger force, 10 g [42].

### 4.12. RNA-Seq

For transcriptome sequencing, total RNA was extracted from seeds of both Nip and OsGRP3-overexpression lines. Samples were collected under two conditions: (1) untreated control seeds and (2) seeds that had undergone 15 days of accelerated aging treatment. Three biological replicates were sequenced for each condition.

Total RNA was extracted from samples using the mirVana™ miRNA Isolation Kit (Ambion, Austin, TX, USA). Eukaryotic mRNA was then enriched using magnetic beads with Oligo (dT), followed by mRNA fragmentation and synthesis of first-strand and second-strand cDNA [15]. The double-stranded cDNA was purified, subjected to end repair and A-tailing, and ligated with sequencing adapters. The constructed library was amplified and quality-checked using the Agilent 2100 Bioanalyzer (Agilent Technologies, Santa Clara, CA, USA).

Sequencing was performed on the Illumina HiSeq™ 2500 or HiSeq X Ten platform. Raw sequencing reads were processed through quality filtering to obtain high-quality clean reads. The FPKM (Fragments Per Kilobase of transcript per Million mapped reads) values for each gene were analyzed using the boNIPie2 software (v 1.0.4), while read counts were obtained using eXpress (1.3.1). Data normalization was performed with the estimateSizeFactors function from the DESeq R package (v 4.4.1), and the nbinomTest function was used to calculate p-values and fold changes for differential comparisons. Differentially expressed transcripts were identified with a q-value < 0.01 and a fold change > 2, followed by GO and KEGG enrichment analyses.

All samples were collected at the mature seed stage following the respective treatments. The RNA-seq data generated in this study have been deposited in China National Center for Bioinformation with the accession number subCRA057614.

### 4.13. Localization Analysis of OsGRP3

To analyze the subcellular localization of OsGRP3, we first harvested seeds from stable transgenic plants transformed with pCAMBIA1390-OsGRP3-GFP and pCAMBIA1390-GFP vectors, respectively. The harvested seeds were germinated and cultured at 26 °C for 4 days, after which root organs of the germinated seedlings were collected. Subsequently, GFP fluorescence signals in the root organs were observed under a confocal laser scanning microscope to determine the precise subcellular localization of OsGRP3 [33].

### 4.14. Preparation of OsGRP3 Antibody

The recombinant OsGRP3-GST fusion protein was expressed in E. coli and purified using glutathione agarose beads to serve as the antigen. The purified protein was then used to immunize mice, and the resulting antiserum was collected according to Wang et al. [15].

### 4.15. Western Blot Analysis

Rice leaf samples were ground to a fine powder in liquid nitrogen and promptly transferred to a 1 mL tube. A 100 μL volume of pre-chilled RIPA buffer was added to extract total protein. Four-fold SDS loading buffer was then added to the supernatant to prepare the samples. The prepared samples were loaded for SDS-PAGE gel electrophoresis, followed by membrane transfer. The membrane was placed in blocking buffer for room temperature blocking. Primary antibody was added at a 1:4000 dilution and incubated at room temperature. Secondary antibody was then added at a 1:6000 dilution and incubated at room temperature. The membrane was washed with TBST. Protein detection was performed by development. Western blot analyses in this study were performed using a commercially available anti-GFP antibody (Abcam, Cambridge, UK, ab290) and an anti-Flag antibody (Sigma-Aldrich, MI, USA, F1804).

### 4.16. RNA Extraction and RT-qPCR

Total RNA was isolated from various rice organs, including root, stem, leaf, seed, and caryopsis, using TRIzol reagent (Thermo Scientific, Waltham, MA, USA, 15596018CN), following the manufacturer’s protocol [15]. Approximately 1 μg of total RNA was used for reverse transcription into first-strand cDNA with the Maxima H Minus First Strand cDNA Synthesis Kit (Thermo Scientific, USA, K1691). Quantitative real-time PCR (RT-qPCR) was subsequently performed to analyze gene expression levels, which were normalized using the rice OsActin2 and PP2A gene as an internal control (Table S1).

### 4.17. Accession Numbers

Sequence data from this article can be found in the MSU/TAIR database under accession numbers AtGRP7 (AT2G21660); AtGRP3S (AT2G05380); AtGRP2B (AT2G21060); AtGRP3 (AT2G05520); AtGRP4 (AT5G07510); AtGRP5 (AT3G20470); AtGRP6 (AT5G07540); AtGRP8 (AT5G07520); AtGRP9 (AT2G05440); AtGRP23 (AT1G10270); OsGRP1A (LOC_Os12g43600); OsGRP1 (LOC_Os01g68790); OsGRP2 (LOC_Os03g56020); OsGRP3 (LOC_Os03g46770); OsGRP4 (LOC_Os04g33810); OsGRP5(LOC_Os05g13620); OsGRP6 (LOC_Os12g31800); OsGRP162 (LOC_Os03g43990).

### 4.18. Statistical Analysis

All data are presented as the mean ± standard deviation (SD) from at least three independent biological replicates. Statistical significance between two groups was determined using a two-tailed Student’s t-test. For comparisons involving more than two groups, one-way analysis of variance (ANOVA) followed by Tukey’s LSD test was performed. Differences were considered statistically significant at *p* < 0.05. All statistical analyses were conducted using GraphPad Prism version 9.0.

## Data Availability

The original contributions presented in this study are included in the article/Supplementary Material. Further inquiries can be directed to the corresponding authors.

## Supplementary Materials

The following supporting information can be downloaded at: https://www.mdpi.com/article/10.3390/plants15030464/s1.
