# Supplementary material for: RNA-Binding Protein OsGRP3 Positively Regulates Rice Storability
Source: Plants (Basel). 2026 Feb 2;15(3):464. doi: 10.3390/plants15030464 (PMC12899378; doi:10.3390/plants15030464)
Supplement: Supplementary file 1 [file plants-15-00464-s001.zip › supporting data-12.17.pdf]

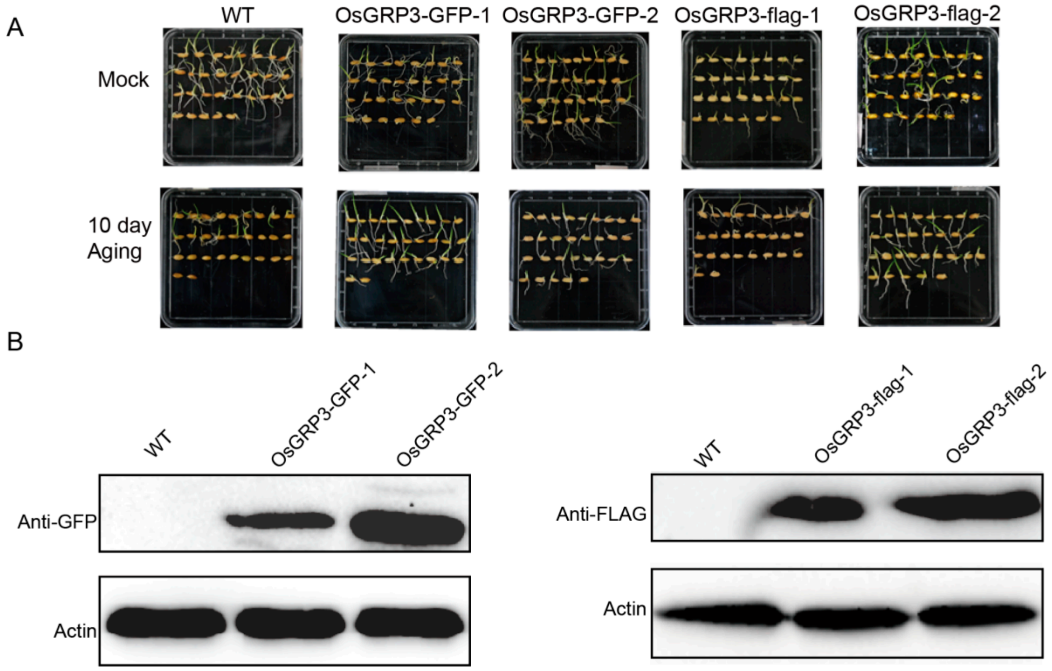

Figure S1. Overexpression of OsGRP3 enhances seed aging tolerance in rice

A, Phenotypic comparison of seed germination in WT and OsGRP3 overexpression lines (*OsGRP3-GFP*, *OsGRP3-flag*) under mock conditions and after 10 days of accelerated aging treatment. Seeds overexpressing OsGRP3 show better germination and seedling growth after aging stress compared to WT.

B, Western blot analysis of WT, *OsGRP3-GFP*, and *OsGRP3-flag* plants using Anti-GFP and Anti-FLAG antibody to detect the OsGRP3 fusion protein, with Actin as the loading control.

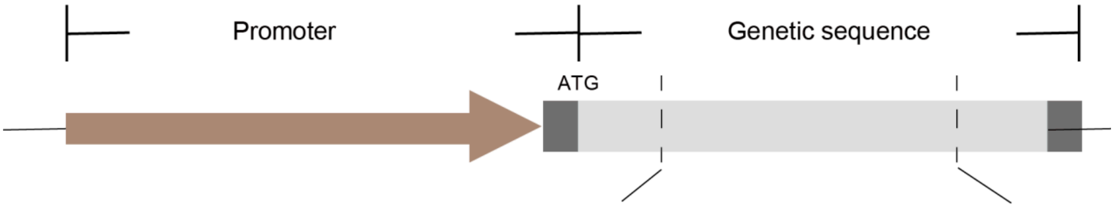

HAP	Aus	Bas	GJ	XI	admix	26460647	26460979
1	138	52	645	732	51	T	C
2			3	544	10	C	T
3	54	19	188	209	18	–	C
4	1	1	1	212	5	–	T
5	1	2	3	12	1	–	–

Figure S2. Haplotype analysis of OsGRP3 among different subspecies

This figure illustrates the structure of a gene region (encompassing the promoter and genetic sequence) along with nucleotide variations at two positions (26460647 and 26460979) across distinct rice subgroups (Aus, Bas, GJ, XI, admix). The promoter region (brown) regulates the transcription of the genetic sequence (light gray, initiated by ATG); the numbers beneath each subgroup denote the number of accessions. The nucleotide variations reflect the

genetic polymorphism at these loci among different rice subgroups.

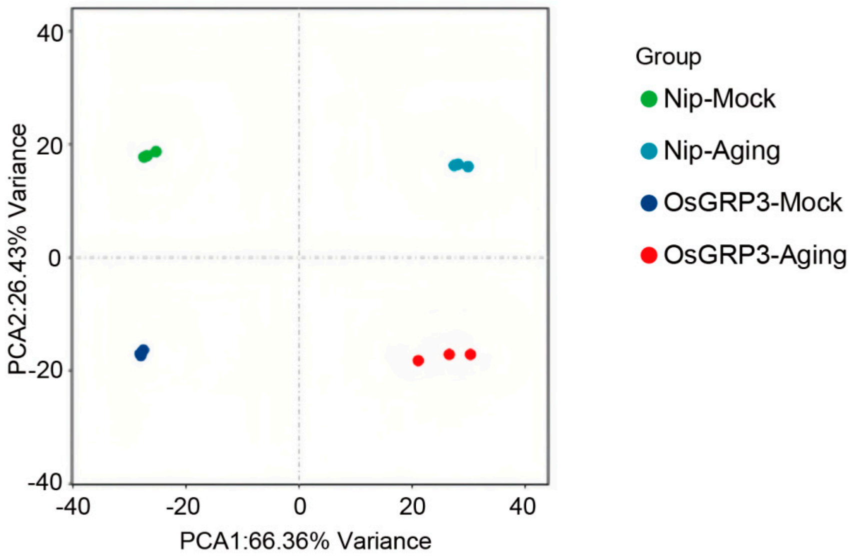

Figure S3. PCA analysis of the rice seed transcriptome

The PCA plot displays transcriptome profiles of the Nipponbare non-aged group (Nip-Mock), Nipponbare aged group (Nip-Aging), OsGRP3-overexpression non-aged group (OsGRP3-Mock), and OsGRP3-overexpression aged group (OsGRP3-Aging). Principal component 1 (PC1) explains 66.36% of the variance, while principal component 2 (PC2) explains 24.43% of the variance.

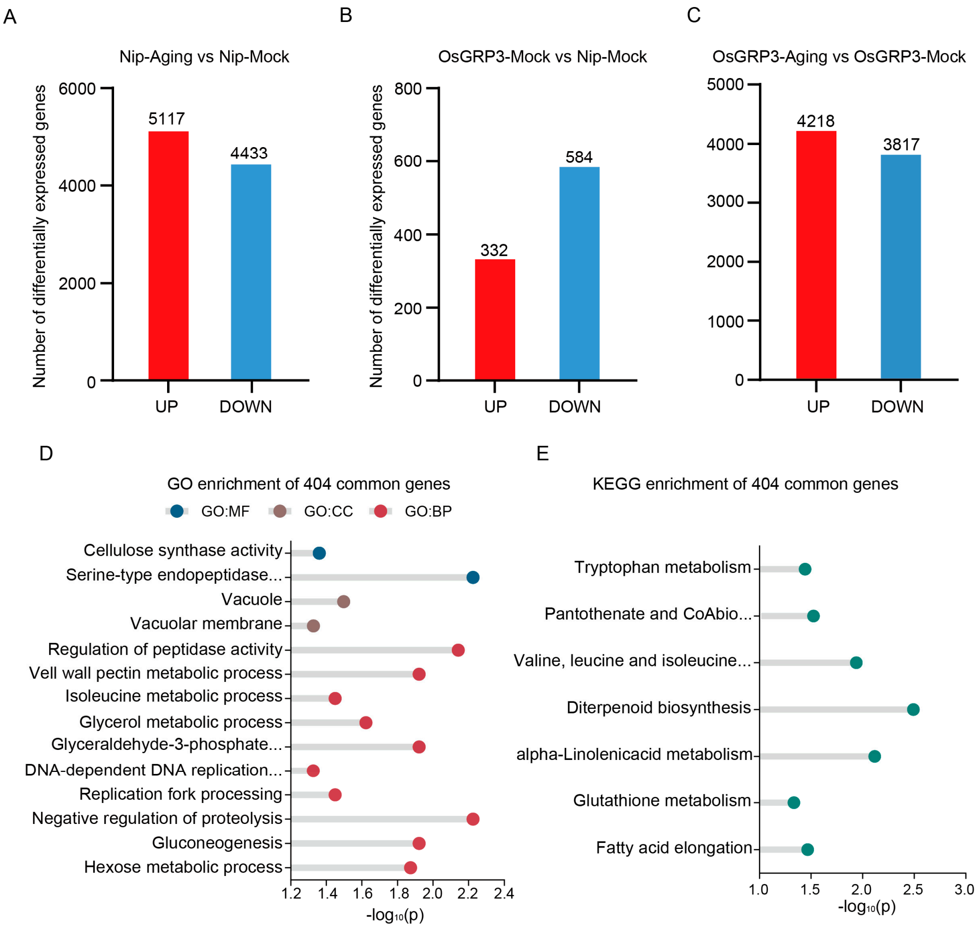

Figure S4. Transcriptional profiling of common differentially expressed genes under aging stress and functional enrichment analysis of 404 common DEGs

A, Number of differentially expressed genes (DEGs) ($|log_2FC| > 1$, $q < 0.01$) between the Nip-Aging and Nip-Mock groups. Red and blue bars represent 5,117 up-regulated and 4,433 down-regulated genes, respectively, indicating that the aging treatment triggers extensive changes in the gene expression profile.

B, Number of DEGs ($|log_2FC| > 1$, $q < 0.01$) between the OsGRP3-Mock and Nip-Mock groups. Red and blue bars represent 332 up-regulated and 584 down-regulated genes, respectively, demonstrating that OsGRP3 overexpression itself alters the transcriptional landscape even in the absence of aging stress.

C, Number of DEGs ($|log_2FC| > 1$, $q < 0.01$) between the OsGRP3-Aging and OsGRP3-Mock groups. Red and blue bars represent 4218 up-regulated and 3817 down-regulated genes, respectively, reflecting the substantial transcriptional differences under both OsGRP3 overexpression and aging treatment.

D, GO enrichment analysis of the 404 common differentially expressed genes, showing significantly enriched terms across three categories: Molecular Function (GO:MF), Cellular Component (GO:CC), and Biological Process (GO:BP). The x-axis represents the $-log_{10}(p)$, with higher values indicating greater enrichment significance.

E, KEGG pathway enrichment analysis of the 404 common differentially expressed genes. The x-axis represents the $-log_{10}(p)$.
